# Resistance a major hindrance to chemotherapy in hepatocellular carcinoma: an insight

**DOI:** 10.1186/s12935-018-0538-7

**Published:** 2018-03-20

**Authors:** K. Lohitesh, Rajdeep Chowdhury, Sudeshna Mukherjee

**Affiliations:** Department of Biological-Sciences, Birla Institute of Technology and Sciences (BITS), Campus, VidyaVihar, Pilani, Rajasthan 333031 India

**Keywords:** Cancer, HCC, Chemoresistance, Metastasis, Apoptosis, Autophagy

## Abstract

Hepatocellular carcinoma (HCC) is one of the leading causes of cancer mortality, accounting for almost 90% of total liver cancer burden. Surgical resection followed by adjuvant and systemic chemotherapy are the most meticulously followed treatment procedures but the complex etiology and high metastatic potential of the disease renders surgical treatment futile in majority of the cases. Another hindrance to the scenario is the acquired resistance to drugs resulting in relapse of the disease. Hence, to provide insights into development of novel therapeutic targets and diagnostic biomarkers, this review focuses on the various molecular mechanisms underlying chemoresistance in HCC. We have provided a comprehensive summary of the various strategies adopted by HCC cells, extending from apoptosis evasion, autophagy activation, drug expulsion to epigenetic transformation as modes of therapy resistance. The role of stem cells in imparting chemoresistance is also discussed. Furthermore, the review also focuses on how this knowledge might be exploited for the development of an effective, prospective therapy against HCC.

## Introduction

Hepatocellular carcinoma is currently considered as a rapidly evolving disease. According to the Globocan report, an estimated 782,451 new liver cancer cases and 745,517 cancer deaths have occurred worldwide in 2012. Also, as per the National Cancer Institute’s Surveillance Epidemiology and End Results (SEER) the relative 5-year survival rate of HCC between 2002 and 2008 has been as low as 15%. The main causative factors contributing to the disease have been chronic alcohol abuse, infection with hepatitis B or hepatitis C virus and food contaminations [1]. As a consequence of such varied etiologies, HCC is a heterogeneous malignancy with complex carcinogenesis. Also despite advances in development of early detection methodologies, the ineffective and expensive procedures available for treatment of HCC pose a challenge for the disease management. In fact, 80% of HCC patients are currently diagnosed at an advanced stage of the disease with a median survival of 6–8 months only. Surgical resection followed by chemotherapy is the most established curative treatment for HCC. However, operating on the liver can be both complicated and unachievable due to size and distribution of the tumor in the liver, blood vessels and other vital organs. Also, complete surgical removal is mostly not possible for more than two-third of HCC patients where the disease have already metastasized and the patients are at an advanced stage [2–4]. Current treatment procedures mainly include cryosurgery, radiofrequency ablation and embolization but they are mostly palliative approaches without much success rate [5]. Moreover, post-surgery recurrence of the tumor has been a major issue for more than 90% of HCC patients. This has forced to shift the treatment regime towards systemic chemotherapy. Drugs that are used in HCC as monotherapy are listed in Table 1. But currently the use of single agents in therapy is practically non-existent because of their low response. For example, in a large study of doxorubicin, no responses were noted among 109 patients; also among 475 patients who received doxorubicin in various studies, only 16% response rate was documented, with a median survival of 3–4 months only [6]. This led to the evolution of combined regimen drugs. A combination of capecitabine + oxaliplatin + cetuximab showed modest activity only [7]. Among cisplatin-based regimens, the best response rate was obtained with the treatment of PIAF (cisplatin + adriamycin + 5-FU + INF) [8]. More recently, GEMOX (gemcitabine + oxaliplatin) has also been evaluated in a phase-II study, with promising results [9]. Other chemotherapeutic drugs like, sorafenib are also often used to attenuate HCC tumor [10, 11]. But, acquisition of chemoresistance continues to be a major constraint in chemotherapy-based treatment of the disease. An alternative strategy adopted was the administration of chemo-drugs like cisplatin, mitomycin C and doxorubicin through hepatic artery infusion [12]. However, surgical catheter insertion into the hepatic artery and inoperable conditions of the tumor owing to HCC’s high metastatic potential became a limiting factor. Thus despite recent advancements in chemotherapy, HCC still remain a fatal disease. Hence, focus should be reoriented more on unraveling the molecular mechanisms behind chemoresistance with an objective to develop novel therapeutic targets and diagnostic biomarkers.

**Table 1:** List of drugs and their targets used against HCC

S. No.	Name of the drug	Target molecule	Mechanism	Limitation	Year and references
1	Tamoxifen	Antagonist of estrogen receptor	To inhibit P-glycoprotein-mediated drug resistance	Minimum effect	2000 [88]
2	5-Fluoracil	Thymidylate synthase	Incorporated its metabolites into RNA and DNA	Requires co-treatment with leucovorin and methotrexate, to increase the anticancer activity of 5-Fu	2003 [89]
3	Thalidomide	VEGF, inhibits TNF-α synthesis, inhibition of Ikβ kinase activity	Anti-angiogenic activity and immune-modulatory	Fatigue, somnolence, constipation	2003, 2004 [90, 91]
4	Octreotide	Analogue of somatostatin receptors	Anti-tumor effect.	Somatostatin receptor type 2 (SSTR2) was found in some but not all patients with HCC	2004, 2006 [92, 93]
5	Sorafenib	Raf, VEGFR2, VEGFR3, PDGFRs	Inhibits tumor angiogenesis by blocking the activation of the tyrosine kinase receptors	Hypertension, diarrhea, proteinuria, skin-related toxicities, an increased risk for thromboembolism and bleeding events	2006, 2008 [10, 11]
6	Sunitinib	PDGFRs, KIT, RET, and FLT3	Inhibits tumor angiogenesis by blocking the activation of the tyrosine kinase receptors	Modest clinical efficacy	2009 [94]
7	Bevacizumab	VEGF	Blocks VEGF binding to its receptor	Low rate of response, gastrointestinal bleeding, including variceal bleeding	2009 [95]
8	Erlotinib, gefitinib and cetuximab	EGFR	Tyrosine kinase inhibitor, acts on the epidermal growth factor receptor (EGFR)	Minimum effect	2009 [96]
9	Doxorubicin	DNA topoisomerase II inhibitors	Induce histone eviction	The use of single agents in therapy is practically non-existent currently because of its erratic and low response	2013 [97]
10	Cisplatin	DNA	Cross link with purine causes DNA damage and ultimately induces apoptosis	Allergic reactions, gastrointestinal disorders, decrease immunity to infections, kidney problems, hemorrhage	2014 [98]
11	Oxaliplatin	DNA	Binds to guanine and cytosine leading to cross-linking of DNA	Increase autophagy level results in a tumor resistance Reduction of DYRK2 promotes cell proliferation, and resistance to Oxaliplatin	2016 [99]

## Various mechanism of chemoresistance in HCC cells

### Reduced drug uptake, efflux of drugs and drug metabolism

A poor response to pharmacological regimens in the treatment of liver cancer can be due to complex mechanisms like, reduced drug uptake, enhanced drug efflux, intracellular drug metabolism leading to reduced concentrations of active agents, changes in the molecular targets of the drugs and enhanced repair of drug-induced modifications. In some cases, the conjoint enhanced expression of drug efflux pumps like, both MDR1 and MRP2 seems to be a contributing factor towards resistance [13]. Thus in patients with non-surgical HCC the response to chemotherapy has been found to be inversely proportional to the expression of multi-drug transport system. In this context, different transporters with their mode of action are listed in Table 2. Hence, recent therapeutic strategies must involve drug pump antagonist against the resistant cells. Alternate mechanisms employed by the tumor cells to inactivate anti-cancer drugs are over-expression of detoxifying enzymes like phase-I (cytochrome P450) and phase-II enzymes in conjugation with glucuronic acid or with glutathione. Cytochrome P450 enzymes and glutathione S-transferases, along with other enzymes like epoxide hydrolase, phase II xenobiotic metabolizing enzymes are reported to increase cells’ resistance to alkylating agents or drugs like, cisplatin in HCC [14, 15]. Enhanced drug in-activation by uridine glucuronosyltransferase-1A has also been found to be associated with drug resistance in HCC [14, 16]. Thus, a vivid understanding of the intra-cellular biology of chemoresistance is desirable to develop new therapies, which would help to override the resistance acquired by the conventional cytotoxic drugs.

**Table 2:** List of drug transport systems and their status in HCC

S. No.	Transporter	Mode of action	Up-regulated or down-regulated	References
1	MDR1 (ABCB1 or P-glycoprotein), MRP1 (ABCC1), MRP2 (ABCC2), MRP3(ABCC3) and ABCG2	Extrudes unrelated anti-tumoral agents like anthracyclines, taxanes, vinca alkaloids etc. from the cell	Up regulated	[18, 100]
2	OATP1B1 and OATP1B3	Transports anti-tumor drugs inside the cells	Down regulated	[101, 102]
3	SLC28 and SLC29	Uptake of nucleoside derived anticancer drugs	Down regulated	[103]
4	Organic cationic transporter-1 (OCT1)	Uptake of tyrosine kinase inhibitors	Down regulated; hyper-methylation of the SLC22A1 promoter	[104]

### DNA repair pathway aberrations and chemoresistance in HCC

HCC develops in a milieu of underlying chronic inflammation, which promotes DNA damage and chromosomal aberration. It has been observed that HCC deploys DNA repair mechanisms, like stalled DNA replication fork by homologous recombination (HR), base mismatches by mismatch repair (MMR), double-strand break (DSB), non homologous end joining (NHEJ) to become chemoresistant. They can upregulate various enzymes involved in DNA repair mechanisms for acquisition of chemoresistance [17]. However, it is not necessary that the repair pathways are always upregulated in chemoresistant models. Defect in MMR pathway genes can also induce resistance to certain therapeutic agents. In HCC cells, over-expression of MDR-1 is associated with patients having defective MMR genes contributing to drug resistance [18]. A better understanding of the role of DDR pathways in HCC may help us to develop novel strategies for treatment or prevention of HCC. Various DNA repair enzymes, which are involved in chemoresistance, are listed in Table 3; these can be exploited in a judicious way to develop novel therapeutic strategies.

**Table 3:** List of DNA repair enzymes de-regulated in HCC

S .No.	DNA repair enzyme	Therapy given	Repair mechanism involved	Reference
1	ERCC-1	Platinum based anti-cancer agents	Nucleotide excision repair (NER)	[105]
2	Flap endonucleases (FENs)	Cisplatin	Nucleotide excision repair (NER) and Base excision repair (BER)	[106]
3	Chk2	Paclitaxel	DNA damage checkpoint	[107]
4	ATM signaling	Sorafenib	DNA damage checkpoint	[108]
5	Apurinic/apyrimidinic endonuclease (APE1)	Irradiation	Base excision repair (BER)	[109]

### Impairment of the apoptotic machinery and associated chemoresistance in HCC

Apoptosis is a highly conserved phenomenon contributing to tissue homeostasis by targeted elimination of single cells without disrupting the functionality of the respective tissue. Cancer strikes by developing resistance against cell death signaling pathways, like apoptosis upon failure of immune surveillance. A similar aspect underlies the poor responsiveness of HCC towards chemotherapy. Upon exposure to cytotoxic agents, HCC cells down regulate death receptor CD95 and upregulate CD95-ligand (FasL) [19–22]. The defect in down-stream Fas-associated death domain (FADD) signaling also potentially contributes to the chemoresistance in HCC [23, 24]. It has further been observed that the anti-apoptotic protein-brain and reproductive organ-expressed protein (BRE), can bind to tumor necrosis factor (TNF) receptor-1 and Fas conferring the ability of chemoresistance to HCC cells by inhibiting death receptor induced apoptosis [25, 26]. FLICE inhibitory protein (c-FLIP), a recently identified intracellular inhibitor of caspase-8 activation that can potentially inhibit death signaling mediated by various death receptors, like Fas, TNF-receptor (TNF-R), and TNF-related apoptosis-inducing ligand receptors (TRAIL-Rs) and by NFĸB activation, was found to be constitutively expressed in HCC cell lines and over-expressed in human HCC tissues. An over-expression of c-FLIP was associated with shorter recurrence free survival time in HCC patients [27, 28]. A resistance towards TRAIL induced apoptosis is also reported in HCC by virtue of loss of TRAIL receptor and over activation of NF-κB. Activation of NF-κB, and nuclear localization of NF-κB, p65, p50 and p52 subunits is often found to be associated with cell proliferation and resistance in HCC [29]. Furthermore, Mcl-1, an anti-apoptotic member of the Bcl-2 family, is over-expressed in HCC, which contributes to the malignant phenotype and resistance towards apoptosis and chemotherapeutics. On a similar note, over-expression of Bcl-2 and Bcl-xL has been found in HCC cell lines and is associated with resistance to paclitaxel [30]. An over-expression of a specific set of microRNA, like, miR23a that allow the escape from TGF-β-induced apoptosis has also been observed in HCC [31]. Additionally, the presence of abnormally functioning p53 is also a common finding in many drug-resistant tumor cells and HCC is no exception. It is reported that p53 contributes to cell survival and chemoresistance in HCC under nutrient-deprived conditions by enhancing autophagy [32]. Also, gain of function (GOF) mutations in p53 are reported to impart resistance to doxorubicin and paclitaxel in HCC cells [33]. Overall apoptosis resistance in HCC is the outcome of varied molecular alterations and further research needs to be channelized in this direction to trace the links to individual pathways regulating apoptosis in HCC.

### Activation of cell survival signaling and evasion of chemoresistance in HCC

In conjunction with de-regulation of apoptosis, HCC cells simultaneously trigger multiple survival signaling pathways to evade the physicians armory and maintain neoplastic progression. For example constitutive activation of Insulin-like growth factor (IGF) signaling pathway is an important contributor to drug resistance in HCC against sunitinib [34]. Various other growth receptors like epidermal growth factor receptor (EGFR) and connective tissue growth factor (CTGF) are also reported to be upregulated and involved in proliferation and drug resistance of HCC [35, 36]. Also, over-expression of Forkhead box M1 (FoxM1) transcription factor that act as a master regulator of HCC cell growth through regulation of cell cycle, glycolysis and EMT contributes significantly to the development of chemoresistance in HCC [37]. The multiple proliferation signaling pathways that enhance angiogenesis, drug resistance and cell proliferation facilitating HCC cell growth are summarized in Table 4. A targeted therapy by specific inhibition of alternated signaling cascades can therefore be thought upon as one of the options to decelerate HCC progression.

**Table 4:** Molecular signaling pathways altered in HCC

S. No.	Altered signaling pathways	Relevant molecule	Alteration	Targeted therapies	References
1	Hedgehog	SMO	Activating overexpression	GDC-0449, cyclopomine	2006 [110]
7	Hippo	MST1/2	Down-regulated	–	2009 [111]
8	Hippo	pYAP	Down-regulated	–	2009 [111]
3	Notch	NOTCH1	Overexpression	Gamma secretase	2009 [112]
2	Wnt/beta-catenin	APC	Inactivating mutation	–	2012 [113]
6	PI3K/AKT/mTOR	MTORC1	Up-regulated	Everolimus, rapamycin	2012 [114]

### Autophagy as defense to chemotherapeutic stress

Autophagy is an evolutionarily conserved process through which lysosomal degradation of damaged and superfluous cell components are achieved and recycled back into basic bio-molecules in the cytosol. HCC cells can use autophagy as a cellular defense mechanism upon facing stress conditions like, nutrient deprivation, hypoxia and drug insult. Reports of autophagy activation post oxaliplatin treatment and enhanced oxaliplatin induced cell-death upon autophagy inhibition in HCC, supports the pro-survival role of autophagy in HCC cells [38]. Similar to above, the anticancer effects of two other drugs, like, bevacizumab and sorafenib were enhanced upon autophagy inhibition in HCC [39, 40]. Our study, also shows that autophagy plays a pro-tumorigenic role facilitating EMT in HCC cells [41]. Inhibition of autophagy by late stage autophagy inhibitors like, chloroquine showed a marked suppression of liver tumor in vivo studies as well [39]. Furthermore, cisplatin treatment is known to trigger unfolded protein response (UPR) in HCC cells which subsequently inhibits cell apoptosis through activation of autophagy [42]. Accumulation of phosphorylated p62, a protein that is generally degraded through autophagy, was found to stimulate hepatitis C virus induced tumors in HCC. Concurrently, inhibition of p62 phosphorylation suppressed proliferation and anticancer drug tolerance in HCC [43]. In spite of a spur in research articles demonstrating the role of autophagy in cancer, the exact role of autophagy on tumor cells is still controversial and remains to be further elucidated in hepatocellular carcinoma.

### Cancer stem cells in HCC chemoresistance

The ‘stem cell model’ of carcinogenesis suggests that cancer originates and is maintained by a rare fraction of cells called the cancer stem cells (CSCs) [44, 45]. They are responsible for the growth of neoplastic tissues and are naturally resistant to chemotherapy. In HCC, CSC markers include epithelial cell adhesion molecule (EpCAM), CD133, CD90, CD44, CD24, CD13, and oval cell marker OV6, some of which confer chemoresistance property to them. CD133+ HCC cells are reported to confer chemoresistance via the preferential activation of the Akt and Bcl-2 survival pathway [46]. The EpCAM+ CSCs in HCC also show chemo-resistance against genotoxic agents like, 5-FU [47]. HCC tumor growth and metastasis are thus undoubtedly driven by CSCs leading to transient effects of the current chemotherapies against HCC.

### Evidences for epigenetic regulation of chemoresistance in HCC

The role of epigenetics, has surfaced as an emerging topic in regulation of chemoresistance in the last decade. Amongst the epigenetic modifications, DNA methylation has garnered a great deal of attention recently in terms of development of chemoresistance. HCC tumors exhibit specific DNA methylation signatures that can be associated with tumor progression implying the potential benefit for their identification and subsequent targeting. Hernandez-Vargas et al. were able to identify a set of hypermethylated gene promoters (APC, RASSFIA, CDKN2A and FZD7) which could differentiate HCC tumors from paired surrounding control liver tissues. Specifically, promoter methylation of DNMT1was also found to be significantly associated with poor prognosis [48]. Song et al. further analyzed DNA methylation pattern in HCC tissues from 27 HCC patients, and observed significant enrichment of promoter methylation in various genes involved in cell death and cancer; the top five of them were genes like, BMP4, CDKN2A, GSTP1, and NFATC1 [49]. Also, another study showed that transcriptional repression of miR-193a-3p promoter through hyper methylation is involved in 5-FU resistance in HCC cells [50]. Hence, suppression of DNA methylation was capable of enhancing the sensitivity of HCC cells to 5-fluorouracil (5-FU). Though, 5-aza-2′-deoxycytidine (5-aza-dC), a specific DNA methylation inhibitor alone, did not induce cell death in vitro; however, a combination of 5-aza-dC with 5-FU showed a reduction in cell viability and induction of apoptosis to a greater degree than with 5-FU only [51]. However, in a clinical study with low dose decitabine (5-Aza-2′-deoxycytidine), substantial remission and favorable toxicity profiles were obtained in patients with advanced HCC [52]. Evaluation of another epigenetic aspect- histone methylation has been less explored and correlated with clinico-pathlogical features in HCC. However, high levels of trimethylated histone H3 lysine 4 (H3K4me3), a transcriptional suppressive signature was found to be associated with poor survival, prognosis and aggressive tumor features in HCC [53]. Also, subunits of polycomb repressive complexes-2 (PRC-2)-SUZ12 and EZH2 were found to impart distinct roles during HCC pathogenesis. Over-expression of EZH2 was consistently observed in advanced HCC [54] while in, chronic HBV infection, the HBx protein was found to modulate SUZ12 protein levels, that maintained “stemness” of a sub-population of hepatocyte cells thus contributing to drug refractoriness [55]. Similar to altered DNA methylation and histone modification patterns, aberrant miRNA expression profiles are also linked to drug resistance and liver progression. A list of miRNAs commonly de-regulated in HCC is shown in Table 5. Another sub-population of non-coding RNAs (ncRNAs), the long non-coding RNAs (lncRNAs) have emerged as critical regulators of HCC drug sensitivity thus offering a novel exciting opportunity to treat HCC. LncRNA HOTAIR (HOX antisense intergenic RNA) and MALAT-1 (metastasis-associated lung adenocarcinoma transcript 1) were found to be upregulated in large cohorts of HCC patients and their suppression increased the chemotherapeutic sensitivity of HCC cells to cisplatin and doxorubicin [56, 57]. Alongside DNA methylation inhibitors, HDAC inhibitors have also been investigated in preclinical and clinical studies in HCC. Belinostat, a deacetylase inhibitor was found to stabilize un-resectable advanced HCC [58, 59]. However, more in-depth understanding of epigenetic alterations is required to gain more insights into the in vivo determinants of responses to epigenetic drugs in HCC.

**Table 5:** List of miRNAs de-regulated in HCC

S. No.	MiRNAs deregulated in HCC	Mechanism	Expression Level	Reference
1	miR-21	Potential biomarker for early stage HCC diagnosis	Up-regulated	[115]
2	miR-338-3p	Suppresses HCC cell invasion by inhibiting metalloproteinase (MMP-9)	Down-regulated	[116]
3	miR-122	Inhibits cycle cyclins & reduces MDR expression	Down-regulated	[117]
4	miR-181& let-7	IL-6 and twist-regulated miRNA expression	Up-regulated	[118]
5	miR-193a-3p	Affects DNA methylation state	Up-regulated	[119]
6	miR-199a/b-3p	Targets mTOR and c-met	Down-regulated	[120]
7	miR-210	Targets apoptosis-inducing factor, mitochondrion-associated, 3 (AIFM3) in hypoxic HCC	Down-regulated	[121]
8	miR-494	Reduces the expression of PTEN but increases PI3 K and p-Akt expression	Up-regulated	[122]
9	miR-1180	Activates NF-κB pathway by downregulating its negative regulators	Up-regulated	[123]
10	miR-122	Up-regulates IGF-1R that contribute to activation of RAS/RAF/ERK signaling which is associated with drug resistance	Down-regulated	[124]

### Ribosome biogenesis as HCC resistance mechanism

The cancer cells for prolonged survival can employ excessive ribosome biogenesis and translation initiation. Recently, RACK1, the receptor for activated C-kinase 1, a component of the 40S subunit of ribosome, was found to be upregulated in HCC and contribute to chemoresistance in vitro and in vivo as well. The preferential translation involved in growth and survival was promoted by ribosomal RACK1 coupled with PKCβII by phosphorylating eIF4E. Inhibition of PKCβII or depletion of eIF4E abolished RACK1-mediated resistance in HCC [60]. These results imply that RACK1 may function as a factor promoting chemoresistance in HCC and targeting RACK1 can be an efficacious strategy for HCC cure.

### Role of telomerase in HCC chemoresistance

The enzyme telomerase is activated in many malignant tumors; i It is known to bestow anti-apoptotic and chemoresistant properties to cancer cells. In accordance to above, it was observed that siRNA against human telomerase reverse transcriptase (hTERT) and cisplatin therapy act synergistically in suppression of HCC progression compared to individual therapy [61]. Low-dose of cisplatin also activated telomerase activity in SMMC7721 human HCC cell line. NF-κB has been reported to be responsible for cisplatin-induced activation of the telomerase in a dose dependent manner. It was thus observed that upregulation of hTERT expression by cisplatin is NF-κB-dependent which contributes to chemotherapy resistance in HCC cells [62]. Additionally, translocation of telomerase into mitochondria can prevent intrinsic pathway of apoptosis under chemotherapeutic stress. Cisplatin-resistant SK-Hep1 cells showed increased translocation of hTERT to mitochondria resulting in decrease of apoptosis. Mitochondrial translocation of hTERT reduced mtDNA damage though the telomere length of chemoresistant cells was shortened. Drug-resistant HCC cells can thus escape from apoptosis through hTERT-mediated mitochondrial protection [63].

### Topoisomerases and HCC chemoresistance

Human DNA topoisomerases are essential enzymes that play a central role in DNA duplex maintenance. In many cancers, aberrant expression of topoisomerase2A (TOP2A) has been reported and is considered to be a valuable prognostic marker for tumor advancements, recurrences and predictor of poor survival [64, 65]. Elevated protein score of TOP2A has also been correlated with non-responsiveness to chemotherapy in in vitro doxorubicin resistant HCC models [66]. Another report further shows a copy number gain in TOP2A locus in doxorubicin resistant HCC cells [67]. Strikingly, the topoisomerase transfected cell lines were around five–tenfold more resistant to cisplatin and other alkylating drugs endorsing its role in drug resistance. As expected, hence combination of Tirapazamine (TPZ), a new anti-cancer drug with Topoisomerase I inhibitors exhibited synergistic cytotoxicity and induced significant apoptosis in several HCC cell types [68]. Targeting topoisomerases can thus be an appropriate strategy for HCC patients who are resistant to conventional cytotoxic therapy.

### Altered lipid metabolism

Cancer cells are able to synthesize lipids in a manner similar to embryonic tissues, and an altered lipid metabolism has often been linked to cancer pathogenesis. For example, stearoyl-coA de-saturase (SCD), a rate limiting enzyme and an essential regulator of lipid homeostasis in the liver is strongly over expressed in HCC. Interestingly, administration of 5-FU also resulted in a time dependent, upregulation of SCD through PI3K and JNK-mediated pathways [69]. While, suppression of SCD by genetic or pharmacologic strategies resulted in increased sensitivity to chemotherapy-induced apoptosis suggesting that SCD play a pro-survival role in HCC [69]. Similarly, another enzyme involved in lipid metabolism- carbonyl reductase1 (CBR1), known to protect cells against lipid peroxidation, promoted chemoresistance in HCC through induction of the master transcription regulator and angiogenesis promoter-HIF-1α [70]. Furthermore, isolated mitochondria from HCC with increased cholesterol levels were resistant to release of cytochrome c or Smac/DIABLO in response to various apoptotic stimuli [71]. Thus, altered lipid metabolism can emerge as a novel therapeutic niche in HCC therapy.

### Tumor microenvironment

**Fig. 1:**
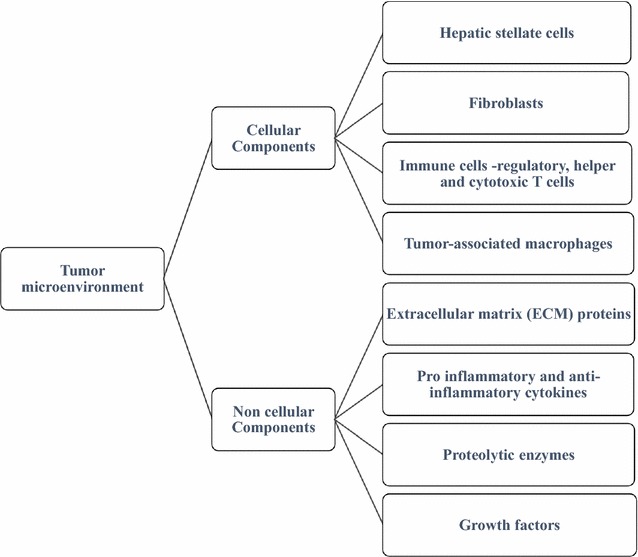
The critical cellular and non-cellular components of HCC tumor microenvironment

Tumor microenvironment (TME) plays a crucial role in HCC development and maintenance. The critical cellular and non-cellular components of HCC tumor microenvironment are showed in Fig. 1 and their functions are summarized in Table 6 [72]. Unlike normal fibroblasts, cancer-associated fibroblasts (CAFs) are the most abundant type of connective tissue present in the TME of multiple cancer cell types [73, 74]. CAFs play an important role in the HCC microenvironment as most liver cancers are derived from fibrosis and cirrhosis. Chuang et al. in 2012 and 2013 showed that co-culture of CAFs with HCC can enhance proliferation, migration, and invasion by altering the transcriptome of HCC cells [75, 76]. The CAF cells can specifically upregulate pro-inflammatory cytokines like CCL2, CCL26, IL6, and LOXL2, which are correlated with proliferation, invasion and angiogenesis of HCC cells [77]. Another key component of HCC tumor microenvironment is the hepatic stellate cells (HSCs), which are generally involved in the process of liver regeneration mostly in case of injuries [78]. However, in addition to its regenerative property, HSCs can exhibit liver carcinogenesis promoting functions as well. They can secrete growth factors and cytokines like, HGF and IL-6 and can engage in a reciprocal crosstalk with HCC cells [79]. Activated HSCs can promote the migration, proliferation and resistance in HCC cells through modulation of TGF-β signaling [80]. Since HSCs are actively involved in tumor progression, targeting HSCs may represent a prospective therapeutic strategy in HCC. Furthermore, immune cells like, CD4 T cells (Treg) and myeloid-derived suppressor cells (MDSCs), which are also part of TME have been implicated in promoting HCC tumorigenesis [81]. Tumor-associated macrophages (TAMs) can also affect HCC tumor progression through NF-κB, STAT-3, and HIF-1 signaling [81]. Further studies are however required to better understand the function of immune cells in HCC TME for enhancement of immunotherapeutic strategies. Table 7 shows some of the tumor microenvironment targeting drugs that are now under investigation for HCC treatment.

**Table 6:** List of various components of HCC tumor microenvironment and their role

S. No.	Cellular and non cellular components	Role in tumor microenvironment	Effect of various components	References
1	Tumor associated macrophages (TAMs)	Tumor development by impairing cytotoxic CD8+ T cell mediated immune responses	Chemo resistance in HCC.	[125]
2	Cancer associated fibroblasts (CAFs)	Produces growth factors like hepatocyte growth factor (HGF), members of the epidermal growth factor (EGF), fibroblast growth factor (FGF) and members of Wnt families, and cytokines, such as stromal-derived factor (SDF)-1α and IL-6	Chemo resistance in HCC	[126, 127]
3	Matrix metalloproteinases (MMPs)	Causes tissue remodeling, inflammation, tumor cell growth and metastasis in many cancers. MMP-2,-9, and -14 activate TGF-β1, which reciprocally activates MMP. Upregulation of MMP-9 is connected with provocation of PI3 K/PTEN/AKT/mTOR pathways in human HCCs. MMPs also inhibit apoptosis signaling in cancer cells	Chemo resistance in HCC	[128, 129]
4	Immune cells	CD8^+^ T cells, NK cells are fooled by cancer cells as an immune evasion mechanism	Poor prognosis in HCC patients	[130, 131]
5	Kupffer cells	Upon activation secrete excessive level of osteopontin	Chemo resistance of cisplatin in small cell lung cancer. It can be postulated to play a role in Hepatocellular carcinoma too	[132]
6	Hepatic stellate cells (HSCs)	Produce collagen in the liver. They get activated upon liver damage and undergo phenotypic changes leading liver fibrosis. They secrete hepatocyte growth factor	Chemo resistance in HCC	[133]

**Table 7:** Drugs targeting TME in clinical trials for HCC treatment

S. No.	Drug	Molecular targets	Phases of clinical trial	Year and references
1	Sibrotuzumab	FAPs	I	2003 [134]
2	PI-88	HPR	II	2009 [135]
3	Selumetinib	MEK	II	2011 [136]
4	Brivanib	VEGFR, PDGFR	III	2013 [137]
5	Lilifanib	VEGFR	III	2013 [138]
6	Axitinib	VEGFR	II	2015 [139]
7	Galuniserib	TGF-β	I	2015 [140]

### Targeted therapy against HCC

For many years, the foundation of HCC treatment has been surgery and non-targeted chemotherapy with conventional drugs like, cisplatin, as discussed earlier. However, over the last decade, partly because of advancements in genomic technologies, targeted therapies with drugs that home into specific cancer cell types and bring about molecular changes have cemented their place as standard treatment for many cancers, HCC is no exception. Sorafenib, a molecular multi-kinase inhibitor has currently been the drug of choice for HCC and is the first molecular targeted agent that has shown survival benefits in HCC patients. However, use of sorafenib has also been hindered by acquisition of resistance [82]; Gao et al. found that elevated fibroblast growth factor 19 (FGF19) expression or hyper-activation of FGF19/FGFR4 signaling was essential for sorafenib resistance in HCC [83]. Hence blocking of FGF19/FGFR4 axis by ponatinib, a third-generation tyrosine–kinase inhibitor, could overcome the resistance of HCC cells to sorafenib [83]. Further, anti-angiogenic drugs like, sunitinib, linifanib and brivanib were tried as independent therapy in HCC but they failed to prove their non-inferiority over sorafenib [84]. Also, HCC patients who are intolerant to sorafenib or have high expression of cellular mesenchymal–epithelial transition factor (c-MET) were found to benefit from Tivantinib therapy, a highly selective inhibitor of c-MET receptor tyrosine kinase [85, 86]. Furthermore, progress in immunotherapy has provided new dimensions to HCC therapy as well. Immune tolerance in HCC is mediated through decreased co-stimulatory signaling that results in immuno-suppression. Hence, anti-human cytotoxic T-lymphocyte antigen 4 (CTLA-4) and anti-programmed death 1 (PD-1) monotherapy have been utilized in HCC treatment but with limited success. Some of the immunomodulators now under investigation for HCC treatment listed in Table 8. Another rapidly emerging immunotherapy approach has been chimeric antigen receptor-engineered (CAR)-T cell therapies that have already demonstrated efficacy against hematologic malignancies. CART technology utilizes the antitumor activity of “domesticated” T cells that have been engineered to express cancer specific antigen-targeted-receptor to treat malignant tumors [87]. However, the major challenge for CAR-T cell therapy in HCC has been the selection of specific antigen to differentiate tumor from normal tissue to prevent off-target effects. The antigens that have till date been used as targets in CAR-T therapy for HCC are mentioned in Table 9. While therapeutic benefit has been achieved in early clinical trials with CAR-T therapy, the use of this therapy is still at its infancy and is limited due to either unique properties of HCC, off-target effects, lack of specific tumor antigens in HCC or cost of production [87].

**Table 8:** Immune-modulators in clinical trials for HCC treatment

S. No.	Drug	Molecular targets	Phases of clinical trial	Year and references
1	Tremelimumab	CTLA-4	II	2013 [141]
2	Icaritin	IL-6/Jak2/Stat3	II	2015 [142]
3	Lenalidomide	TNF-α, interferon γ, IL-6, IL-10, and IL-12.	II	2015 [143]
4	Codrituzumab	Glypican-3	II	2016 [144]
5	Nivolumab	PD-1	I	2016 [145]
6	Ipilimumab	CTLA-4	II	2017 [146]
7	Tasquinimod	Protein S100A9	II	2017 [147]

**Table 9:** Application of CAR-T cells for HCC

S. No.	Antigen	Gene transfer vehicle	Phases of clinical trial	Year (Clinicaltrials.gov identifier or reference)
1	Epidermal growth factor receptor	Lentivirus	I/II	2013 [148]
2	Mucin-1	–	I/II	2015 (NCT02587689)
3	CD133	Retrovirus	I	2015 (NCT02541370)
4	Carcinoembryonic antigen	Retrovirus	Preclinical	2016 [149]
5	Epithelial cell adhesion molecule	–	I/II	2016 (NCT02729493)
6	Glypican-3	–	I/II	2016 (NCT02723942)

## Conclusion

**Fig. 2:**
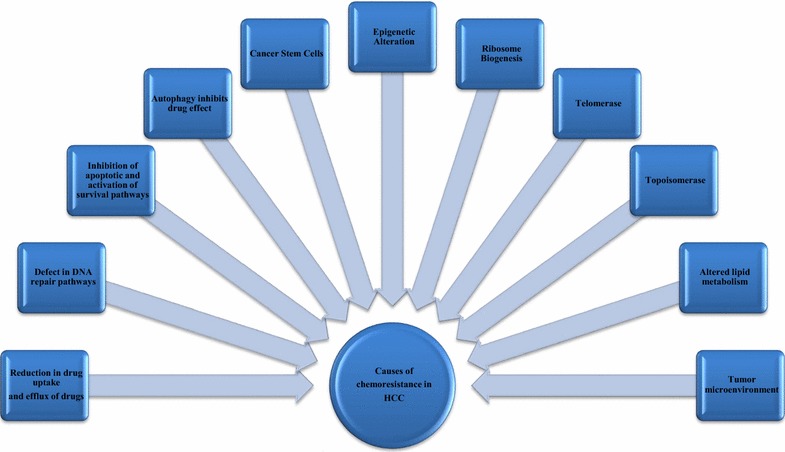
Potential molecular mechanisms that regulate chemoresistance in Hepatocellular Carcinoma

We believe that drug companies are currently fighting a losing battle against advanced HCC tumors. The various chemotherapeutic drugs currently in use against HCC have shown strong chemoresistance providing a massive setback to therapeutic regimens targeting HCC. Also, liver cancer patients generally have a poor tolerance to chemotherapy due to liver dysfunction. Characterization of molecular strategies underlying chemoresistance is hence essentially needed to identify appropriate targets to effectively sensitize these resistant cells. However, designing such strategies are by no means easy. Till date development of targeted drugs has not yet improved the outcome much significantly. This might be attributed to various factors extending from apoptosis evasion, stem cell activation, enhanced DNA repair, topoisomerase activation, lack of proper targets for immunotherapy to dynamic changes in TME and others, as discussed in this literature (Fig. 2). These factors either independently or in unison contribute to emergence of refractoriness to drug therapy in HCC. To compound the scenario, therapeutic effectiveness may also vary depending on patient properties and at various stages of tumor development. Hence, formulating a unified molecular targeted therapeutic strategy for all HCC patients is unlikely to succeed. Therefore, future therapies targeting HCC should be based on combination of context and stage dependent molecular targeted drugs against resistance with or without conventional drugs to successfully treat HCC.
